# Determination of pore size distribution at the cell-hydrogel interface

**DOI:** 10.1186/1477-3155-9-24

**Published:** 2011-05-27

**Authors:** Aldo Leal-Egaña, Ulf-Dietrich Braumann, Aránzazu Díaz-Cuenca, Marcin Nowicki, Augustinus Bader

**Affiliations:** 1 Department of Cell Technology and Applied Stem Cell Biology, Biotechnology and Biomedicine Centre (BBZ), University of Leipzig. Deutscher Platz 5, 04103, Leipzig, Germany; 2 Institute for Medical Informatics, Statistics, and Epidemiology (IMISE), University of Leipzig, Härtelstraße 16-18, 04107 Leipzig, Germany; 3 Materials Science Institute of Seville (Spanish National Research Council (CSIC) - University of Seville), Centro de Investigaciones Científicas Isla de la Cartuja, Avda. Americo Vespucio no. 49, 41092 Sevilla, Spain; 4 Networking Research Center on Bioengineering, Biomaterials and Nanomedicine (CIBER-BBN), Spain; 5 Institute of Anatomy, Medicine Faculty, University of Leipzig, Liebigstrasse 13, 04103 Leipzig, Germany

## Abstract

**Background:**

Analyses of the pore size distribution in 3D matrices such as the cell-hydrogel interface are very useful when studying changes and modifications produced as a result of cellular growth and proliferation within the matrix, as pore size distribution plays an important role in the signaling and microenvironment stimuli imparted to the cells. However, the majority of the methods for the assessment of the porosity in biomaterials are not suitable to give quantitative information about the textural properties of these nano-interfaces.

**Findings:**

Here, we report a methodology for determining pore size distribution at the cell-hydrogel interface, and the depth of the matrix modified by cell growth by entrapped HepG_2 _cells in microcapsules made of 0.8% and 1.4% w/v alginate. The method is based on the estimation of the shortest distance between two points of the fibril-like network hydrogel structures using image analysis of TEM pictures. Values of pore size distribution determined using the presented method and those obtained by nitrogen physisorption measurements were compared, showing good agreement. A combination of these methodologies and a study of the cell-hydrogel interface at various cell culture times showed that after three days of culture, HepG_2 _cells growing in hydrogels composed of 0.8% w/v alginate had more coarse of pores at depths up to 40 nm inwards (a phenomenon most notable in the first 20 nm from the interface). This coarsening phenomenon was weakly observed in the case of cells cultured in hydrogels composed of 1.4% w/v alginate.

**Conclusions:**

The method purposed in this paper allows us to obtain information about the radial deformation of the hydrogel matrix due to cell growth, and the consequent modification of the pore size distribution pattern surrounding the cells, which are extremely important for a wide spectrum of biotechnological, pharmaceutical and biomedical applications.

## Background

Alginate is a natural polysaccharide, which forms stable three-dimensional (3D) hydrogels upon binding divalent cations such as Ca^2+^, Sr^2+ ^or Ba^2+^. Due to the high immune compatibility, the use of alginate to entrap cells has been widely studied with the purpose of entrapping immortalized and/or transformed cells which could replace malfunctioning tissues of a diseased organ [1]. Besides, alginate microcapsules can be used to test the action of anticancer drugs on malignant cells embedded in a 3D environment (tumour-like microcapsules) [2].

Owing to the enhanced proliferation capacity of immortalized and/or cancer cells, the analysis of modifications of the interface between cell and biomaterial with cell growth is highly desirable. Some methods to characterize the porous structure of the 3D networks have been previously reported, such as mercury intrusion porosimetry [3], nitrogen physisorption [4], and the diffusion kinetics of relevant solutes [5]. Nevertheless, these techniques cannot be applied in the presence of cells, nor do they give information about modifications produced at the cell-biomaterial interface due to cell proliferation.

Owing to the feasibility of obtaining and analyzing high resolution electron microscope images of cryofixed cells embedded in 3D matrices, it is one of the most widely used techniques to analyze textural properties of hydrogels, offering the advantage of simultaneously obtaining information pertaining to both the cells and the material comprising the matrix [6]. Since hydrogels are most commonly formed by networks of randomly interconnected polymers, they form complex microarchitectures of cavities with variable shapes and morphologies. Even though well-defined pore-like structures can be clearly observed with scanning electron microscopy [7], we need to consider other approaches for extracting accurate quantitative three dimensional information of the hydrogel matrix from measurements made in two dimensions.

In this paper we describe a methodology based on automated image processing and analysis of transmission electron microscopy (TEM) images obtained from hydrogels, and its applicability on determining modifications of the pore size distribution at the cell-alginate interface as a result of cell growth.

The method was performed after entrapping the hepatocarcinoma cell line HepG_2_, which represents an example of cells with enhanced proliferative capacity.

## Findings

### Material and methods

#### Electron microscopy images

Transmission Electron Microscopy (TEM) images were obtained with an Electron Microscope (Carl Zeiss EM 10, Germany) according to methods published previously [8]. Briefly, the method is based on the fixation of alginate microcapsules with a 2.5% glutaraldehyde solution (Serva, Germany) dissolved in a buffer solution composed of 9 g/l NaCl (Carl Roth, Germany), 5.55 g/l CaCl_2 _(Merck, Germany) and 10.46 g/l of Mops buffer (Carl Roth, Germany). After overnight fixation (4°C), alginate microcapsules were saturated with 2.0% (w/v) agarose (Carl Roth, Germany), and fixed again with 2.5% glutaraldehyde at 4°C for 1 h. Capsules were rinsed three times for 20 min with the buffer solution. Post-fixation was performed by using 1.0% osmium tetroxide (Merck, Germany) at 4°C (2 × 1h), and posterior embedded in Durcupan (Sigma-Aldrich, Germany). Ultrathin sections were stained with uranyl acetate and lead citrate (Serva, Germany) [8].

The total number of TEM pictures obtained was 72, assuming a random distribution of cells within the alginate capsules.

#### Textural properties of cell-free alginate microcapsules [4]

Measurements were carried out after drying the microcapsules in CO_2 _beyond the critical point. N_2 _adsorption-desorption isotherms were collected using a Micromeritics ASAP2010 gas adsorption analyzer at 77K, after degassing the samples at 298K overnight on a vacuum line. The Brunauer-Emmet-Teller (BET) specific surface area was evaluated using adsorption data in a relative pressure range, 0.05 to 0.2 [9]. Alginate matrix pore size distribution was calculated on the basis of the desorption branches using the Barret-Joyner-Halenda method (BJH) [10].

#### Cell culture

HepG_2 _cells (obtained from the departmental cell bank of the Stem Cell Biology laboratory, University of Leipzig, Germany) were cultivated in DMEM (Biochrom, Germany) supplemented with 15% v/v foetal bovine serum (GIBCO, Scotland), 100 ng/ml sodium pyruvate (Sigma-Aldrich, Germany) and 50µg/ml Gentamycin (PAA laboratories, Austria).

#### Cell encapsulation

HepG_2 _cells were immobilized in 0.8% and 1.4% w/v alginate-CaCl_2 _microcapsules of 500µm diameter according to methods described previously [4,8]. A commercially available encapsulation system (Innotech, IE-50R) with a 250µm nozzle was used. This system produces capsules with a diameter of up to 500µm. In all cases, the initial number of immobilized HepG_2 _per mL alginate was 1.5·10^6 ^(approximately 100 cells per capsule). The viability of the immobilized cells before the process of encapsulation was determined by the Tripan Blue exclusion method (Sigma-Aldrich, UK), where the viability of HepG_2 _reached 95%.

#### Determination of cell and/or aggregates sizes

Analysis of cells and/or aggregates radii was carried out by using the program Axiovision (Carl Zeiss, Germany) after images capture of cells and/or aggregates with an Axiovert HRC camera (Carl Zeiss, Germany) mounted on an inverted microscope (Zeiss Axiovert 200). Analyses of size distribution were carried out with a minimum number of 200 capsules, which were placed in a 4 well plate containing 500 μL media, 0.05% v/v concentration of Calcein A/M (Invitrogen, USA) and 0.25% v/v of Ethidium homodimer I (Invitrogen, USA).

#### Image Analysis

Automated analysis of transmission electron microscopy (TEM) was accomplished using the following protocol: firstly, relatively high image-inherent contrast basically makes automatic image segmentation (alginate vs. cavities) straightforward by applying binarization using a simple thresholding, however, preprocessing is required in order to compensate for local contrast fluctuations, so that image inhomogeneity correction using a high-pass filter [11] was applied. Image noise was removed doing a preserving edge-smoothing using total variation filtering [12]. Additionally, coherence-enhancing shock filtering [13] was done to further intensify all directed alginate structures. Pre-processed TEM images were then partitioned into alginate and cavity segments using binarization. The minimum accepted lumen area was set to approx. 275nm². Measurements of these binary images were performed using an unsigned Euclidean image distance transformation [14] providing for all background pixels a respective shortest distance to the surrounding alginate, thereby obtaining values of relative radii of these cavities. The number of times the same value was repeated is hereafter dubbed the *frequency*. All distance transformation-based measurements were accomplished along the skeleton between two opposite alginate fibrils. Discrete values of radii of the alginate cavities are named in this paper as *relative pore radius *(rpr). For images obtained after cell entrapment, we carried out the protocol described above, followed by correlating measurements of relative pore radii to the perpendicular distance from the interface cell-biomaterial, assuming a maximum distance of 400 nm. This was carried out by delineating the cell contour to generate a mask, which was used as a starting point for measurements, again accomplished based on a computational effective Euclidean distance transform. In order to obtain a distribution of values in percent, rpr between 10 and 70 nm were grouped in a discrete cluster. All image processing was accomplished using the computer algebra system MATHEMATICA^® ^(Wolfram Research, Inc., Urbana-Champaign, Illinois, USA) including the Digital Image package written by Jens-Peer Kuska.

Similar to the measurements of relative pore's radii, after treatment of the images with the procedures described previously, measurements of diameters of the alginate fibrils were carried out by measuring the distance transform masked out along a fibril skeleton. The precision of our method depends of the image resolution, where in case of the pictures used in this paper (obtained with an amplification of 20000X), 1 pixel represents 2.34 nm².

## Results and Discussion

**Figure 1:**
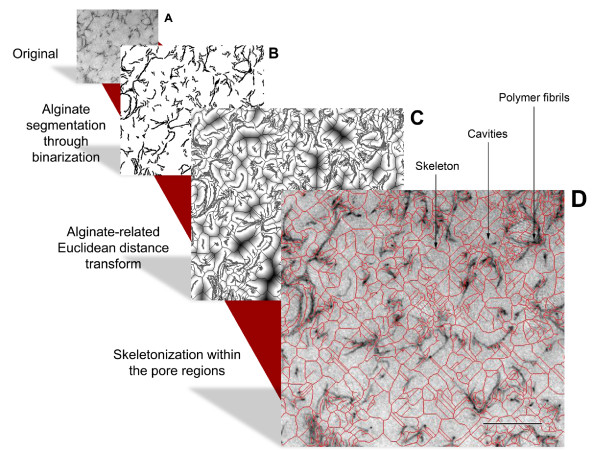
**Illustration of the method to determine pore size distribution developed in this work**. Image A depicts a hydrogel as it is typically observed using transmission electron microscopy. Image B shows the results of the image segmentation after binarization. Image C shows the result of a Euclidean distance transformation. Image D gives an overlay of the pore region image skeleton (red lines) with the original image. Image skeletons are one-pixel wide center axes. They are defined via the set of inner pore pixels. The set is defined via local distance maxima with respect to alginate segments. Scale bar corresponds to 250 nm.

Figure 1 shows 2D images of the matrix nano-architecture of the alginate hydrogel microcapsules. The hydrogel matrix is formed by a network of fibril-like structures which can be identified and discriminated from the surrounding cavities by computational programs. These cavities are named in this paper as *relative pores *(rp). In this work we measured the shortest distance between two opposite points of these fibril-like structures, generating a simulated skeleton, which allowed us to estimate the dimensions of the rp. Half of this distance, named in this paper as the *relative pore radius *(rpr), was used as the criteria for defining the sizes of these cavities. In addition, the frequency in the determination of the same values of rprs was analyzed, with the purpose of studying the pore size distribution. This analysis allows us to compare different concentrations of hydrogels, and the pore size distribution measured with other standardized methods.

**Figure 2:**
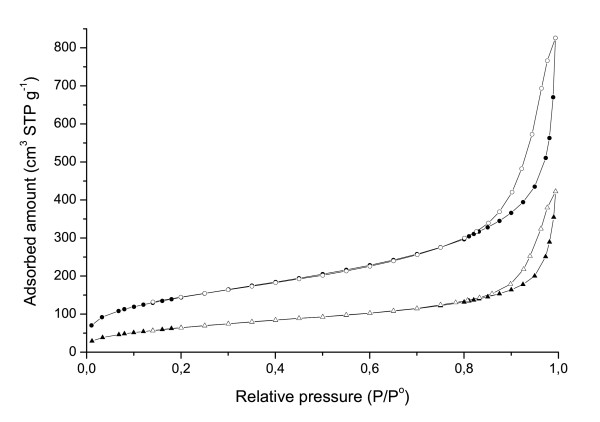
**N_2 _adsorption (black filled symbols) - desorption (unfilled symbols) at 77K isotherms of supercritical CO_2 _dried capsules made of 0.8% (triangles) and 1.4% w/v (circles)**.

To analyze the reliability of our image analysis, the values of the pore size distribution of cell-free microcapsules were compared with those obtained by nitrogen physisorption on dried microcapsules. Although this technique is widely used to measure surface areas in powders and porous networks, it can also provide useful information about pore size in the mesoporous range. The isotherms obtained are presented in Figure 2, and it is possible to observe a similar behaviour to those of type IV and hysteresis type H_3 _according to the IUPAC classification [15], typical for mesoporous solids with strong adsorbent-adsorbate interactions, indicating the presence of large mesopores with a size distribution that continues into the macropore domain (pores > 50 nm). Type H_3 _loops are usually given by adsorbents containing slit-shaped pores in good agreement with the observed network cavities. The adsorption at low relative pressure allowed us to evaluate the specific surface area of the samples by the BET method, assuming a monolayer of N_2 _molecules covering 0.162 nm^2^. Specific surface areas of 245 and 532 m^2^.g^-1 ^have been obtained for capsules made of 0.8% and 1.4% w/v alginate respectively, in a reproducible and well-correlated measurement with the increase in biopolymer material per capsule of similar dimensions (approximately 500 μm in diameter).

Table 1 shows the comparison of the results obtained in microcapsules made of 0.8% and 1.4% w/v alginate, using our image analysis and the N_2_-adsorption-desorption. The good agreement between the results of both methods is clear, with errors lower than 5.0%. The results in Table 1 indicate that alginate hydrogels have a wide distribution of relative pores, with dimensions up to roughly 70nm. Quantities of pores smaller than 10 nm correspond to approximately 50% in the case of alginate 0.8% w/v, and approximately 60% in the case of alginate 1.4% w/v microcapsules, indicating that both matrices seem to be very similar in terms of pore size distribution. Beside the determination of the dimension of the cavities forming the alginate matrix, our methodology allowed us to determine the alginate fibril-like structure width, which is higher in the case of alginate 1.4% than in the capsules made of 0.8% w/v (Table 2).

**Table 1:** Comparison of values of relative pore radius (rpr) determined by N_2 _adsorption-desorption and image analyses in cell-free microcapsules made of 0.8% and 1.4% w/v alginate

Range rpr (nm)	0.8% w/v Alginate		1.4% w/v Alginate	
	**N_2_-adsorption (%)**	**Image analysis (%)**	**N_2_-adsorption (%)**	**Image analysis (%)**

**rpr ≤ 10**	48.9 ± 1.3	49.4 ± 2.3	59.8 ± 1.5	62.8 ± 1.8
**10 < rpr < 20**	15.4 ± 1.1	16.7 ± 2.3	16.1 ± 1.2	19.9 ± 1.1
**20 < rpr < 25**	13.3 ± 1.0	11.1 ± 2.2	8.9 ± 1.0	9.4 ± 1.6
**25 < rpr < 40**	11.3 ± 0.8	9.3 ± 1.9	8.8 ± 0.7	4.7 ± 0.4
**40 < rpr < 70**	9.0 ± 0.5	11.0 ± 0.7	5.3 ± 0.5	2.9 ± 0.3
**70 < rpr**	2.2 ± 0.3	3.0 ± 0.3	1.1 ± 0.2	1.0 ± 0.2

**Table 2:** Comparison of values of fibril-like radii (flr) determined by image analyses in alginate microcapsules made of 0.8% and 1.4% w/v alginate

Range (nm)	0.8% w/v Alginate(%)	1.4% w/v Alginate(%)
**flr ≤ 2.34**	48.7 ± 2.8	30.3 ± 2.4
**2.34 < flr < 4.68**	48.9 ± 3.2	64.0 ± 3.1
**4.68 < flr < 7.02**	2.3 ± 0.3	5.5 ± 0.5
**7.02 < flr < 9.36**	0.1 ± 0.01	0.3 ± 0.1
**9.36 < flr**	0.0 ± 0.0	0.0 ± 0.0

It is important to note that although the hydrogel matrix allows easy diffusion of several nutrients with small molecular weight (e.g. glucose, oxygen), the presence of a high population of pores smaller of 10 nm could restrict the diffusion of some proteins, such as albumin and/or hemoglobin (Stokes radius of 3.1-3.5 nm and 2.4 nm respectively) [16].

It is important to remark that the sensitivity of our method relies on the micrograph image resolution. Thus, the use of image analysis becomes a powerful strategy for the analysis of meso- and nano- porous materials, presenting clear advantages to other strategies for characterization of textural properties of hydrogels.

**Figure 3:**
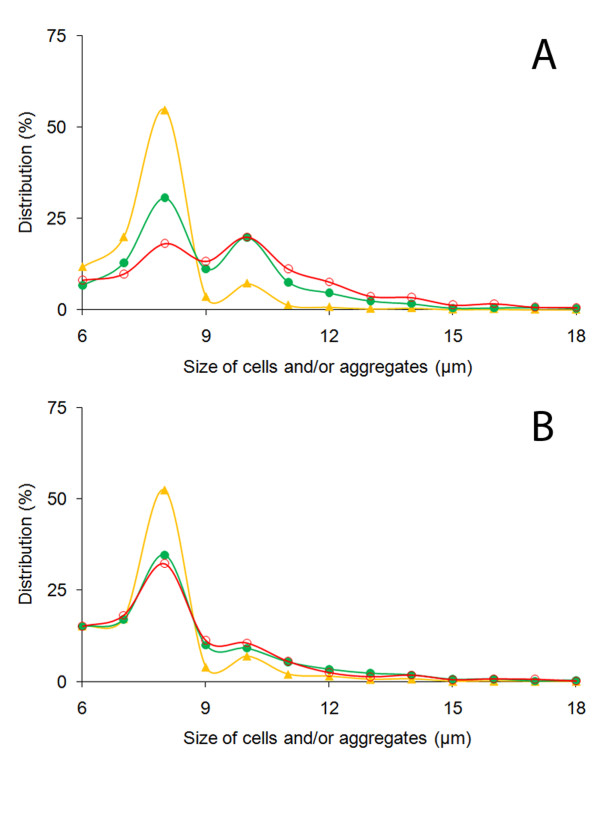
**Sizes of HepG_2 _cell population (individual living cells and aggregates) within microcapsules made of 0.8% (A) and 1.4% w/v (B) alginate, during days 0 (yellow triangles), 3 (filled circles), and 6 (open circles) of culture**.

After characterization of cell-free alginate hydrogel, HepG_2 _cells were entrapped in microcapsules made of 0.8% and 1.4% w/v alginate, and cultured for 6 days, analyzing aggregation and proliferation as increases in the size of single cells and aggregates. Since alginate lacks domains for proteases, entrapped cells cannot migrate into the matrix, generating spherical aggregates after proliferation, which can be analyzed by measuring their diameters [17]. As Figure 3 shows, cells entrapped in 0.8% w/v microcapsules increased their size much more than those immobilized in 1.4% w/v.

Measurements of rp sizes and frequency were carried out on days 0, 3 and 6, in a similar manner to the determinations performed in cell-free hydrogels. These values were correlated with a third parameter measured perpendicularly inwards from the alginate matrix to the cell. This analysis allows us to quantify the extension (depth) to which the cells can modify the material matrix in terms of pore size distribution.

**Figure 4:**
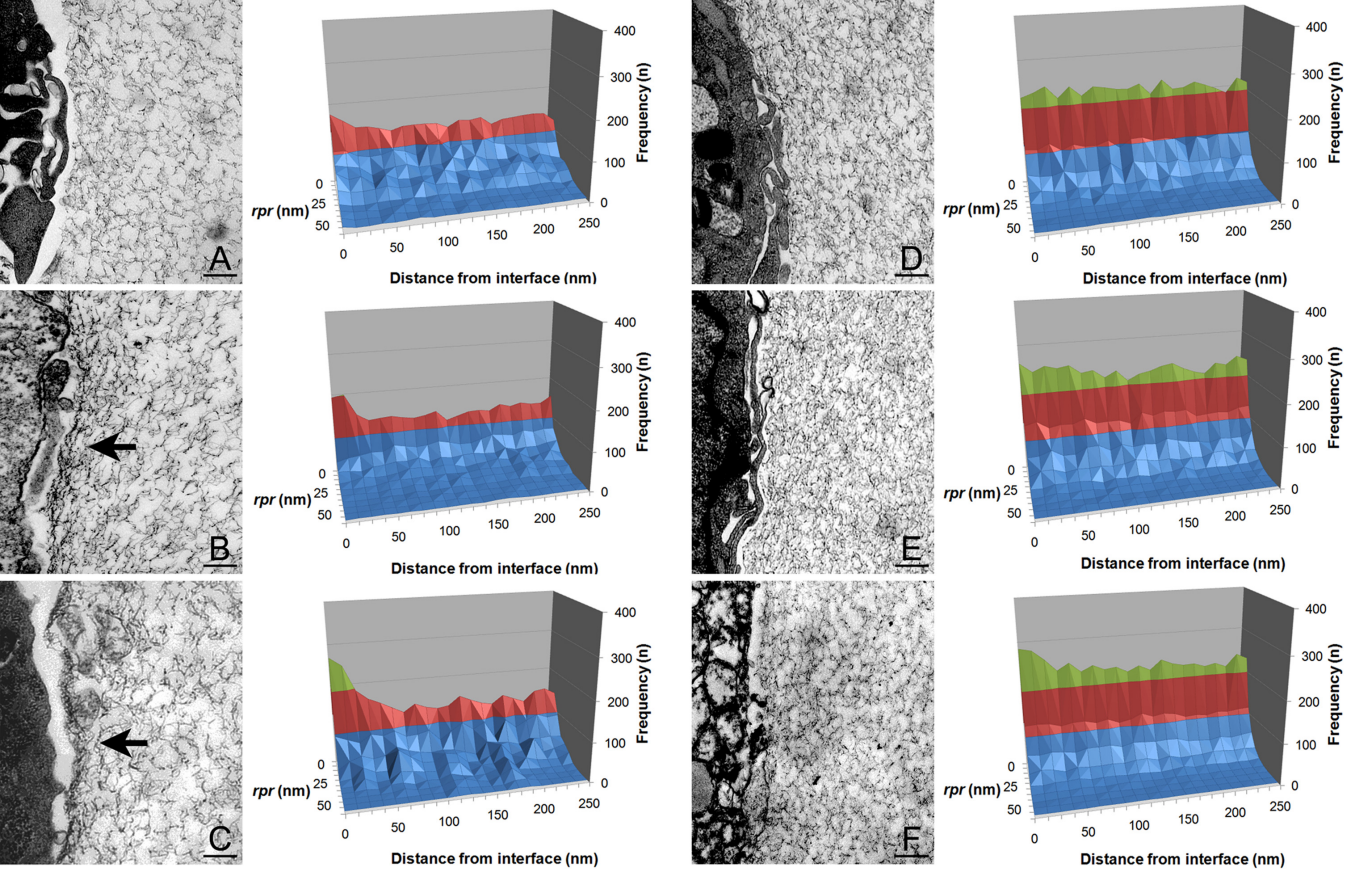
**Analysis of transmission electron microscopy images of the cell biomaterial interface on microcapsules made of 0.8% (A, B, and C) and 1.4% w/v (D, E and F), at day 0 (A and D), 3 (B and E), and 6 (C and F)**. Arrows show zones where clear constriction of the alginate interface due to cell proliferation is observed. Scale bar represents 10 nm.

Our results show significant modifications in the pattern of pore size distribution, mostly observed in case of cells entrapped in hydrogels made of 0.8% alginate, where an increase in the presence of pores smaller than 10 nm was clearly observed (Figure 4). Furthermore, these modifications were observable up to depths of 40 nm from the interface, with the higher coarsening detected within the first 20 nm from the interface. By contrast, only slight deformations were observed in the experiments performed with hydrogels made of 1.4% w/v alginate (approximately 40 nm from the interface), where coarsening of pores seems to be much slower and more homogeneous than in the softer capsules.

The higher resistance of the more highly concentrated hydrogel to mechanical deformation can be explained by increases in both the percentage of pores smaller than 10 nm, and the thickness of the alginate fibril-like structures, due to increased crosslinking of alginate polymer.

According to recent publications, immobilized cells within alginate hydrogels are submitted to compression forces which lead single cells to generate cellular microspheroids [18]. Thus, analyses of radial deformation of the alginate matrix due to cell growth and the consequent modification of the pore size distribution pattern can give us very important information about modulation of rates of molecular diffusion of nutrients/waste products, information which is not only extremely useful for biomedical applications [1], but also for studying the development of primary tumours in tumor-like microcapsules [2,19], as mentioned previously.

It is important to mention that methods for cell fixation can slightly diminish cell size, and therefore a short distance between cells and the material interface can be observed in several cases. Nevertheless, as shown in Figures 1 and 4, this does not affect the pore size distribution and the textural properties of the matrix material, ensuring the reliability of our method. As a final remark, it is important to note that although our methodology has been not tested with other polymers, because it is based on image analysis of TEM pictures, studies of modifications of the cell-hydrogel interface may be possible in different types of hydrogels which maintain their textural properties after fixation.

## Competing interests

The authors declare that they have no competing interests.

## Authors' contributions

ALE conceived and designed the method, performed the experiments and interpreted the data. UDB performed the image analysis and conceived the method. ADC performed the textural analysis and interpreted the data. MN obtained the electron microscopy images. ALE, ADC, and UDB prepared the manuscript. AB and ADC critically revised the intellectual content of the manuscript and gave the final approval of the version to be published. All Authors read and approved the final manuscript.
